# Utility of Estimated Pulse Wave Velocity for Tracking the Arterial Response to Prolonged Sitting

**DOI:** 10.3390/jcdd9120411

**Published:** 2022-11-23

**Authors:** Abdullah Bandar Alansare, Lee Stoner, Osama Eid Aljuhani, Bethany Barone Gibbs

**Affiliations:** 1 Department of Exercise Physiology, College of Sport Sciences and Physical Activity, King Saud University, King Khalid Rd, Riyadh 80200, Saudi Arabia; 2 Department of Sport and Exercise, University of North Carolina, Chapel Hill, NC 27599, USA; 3 Department of Epidemiology, Gillings School of Global Public Health, University of North Carolina, Chapel Hill, NC 27599, USA; 4 Department of Physical Education, College of Sport Sciences and Physical Activity, King Saud University, King Khalid Rd, Riyadh 80200, Saudi Arabia; 5 Department of Epidemiology and Biostatistics, School of Public Health, West Virginia University, Morgantown, WV 26506, USA

**Keywords:** prolonged sitting, sedentary behavior, arterial stiffness, pulse wave velocity, non-invasive measurement

## Abstract

Background: Arterial stiffness, measured by pulse wave velocity (PWV), is a purported mechanism linking sedentary behavior to cardiovascular disease. This secondary analysis compared associations between measured carotid–femoral PWV (cfPWV) and carotid–radial (crPWV) responses to an acute bout of prolonged sitting with mathematically estimated cfPWV (ePWV). Methods: Overweight/obese adults with elevated blood pressure were enrolled (*n* = 25; 42 ± 12 yrs; 64% males). Participants performed an 8 h simulated workday of mostly sitting. cfPWV and crPWV were measured while supine in the morning, midday, and afternoon. ePWV was calculated at the same timepoints using age and seated mean arterial pressure (MAP). Pearson correlation coefficients associated ePWV with cfPWV and crPWV. Generalized linear models separately examined the effects of time on cfPWV, crPWV, and ePWV. Results: ePWV significantly associated with cfPWV and crPWV (*r* = 0.69 and 0.55, respectively; *p* < 0.05) in the morning (baseline). cfPWV significantly increased over time (β = 0.52 ± 0.20 and 0.48 ± 0.21 with and without MAP adjustment, respectively; *p* < 0.05). In contrast, ePWV and crPWV did not significantly increase overtime (β = 0.14 ± 0.09 and 0.25 ± 0.23, respectively; *p* > 0.05). Conclusions: Our results suggest that, although ePWV is associated with cfPWV and crPWV at a fixed timepoint, ePWV responds differently to prolonged sitting and likely does not capture the same acute vascular responses.

## 1. Introduction

Sedentary behaviors (SB), particularly regular prolonged sitting exposures, are a major cardiovascular disease (CVD) risk factor, independent of physical inactivity [1,2]. A proposed mechanism linking SB to CVD is increased aortic arterial stiffness [3,4]. The most widely used measure of aortic arterial stiffness and a strong independent predictor of CVD is carotid–femoral pulse wave velocity (cfPWV) [5,6,7,8]. In support of the biological plausibility for the association between sitting exposure and CVD, we and others have reported temporal increases in cfPWV in response to an acute bout of prolonged sitting [9,10,11]. However, cfPWV measurement, in the context of acute sitting research, poses some limitations. Most importantly, to record the cfPWV measurements in accordance was standard guidelines, participants must be transitioned from the seated to supine posture, interrupting the sitting bout [5]. This postural shift could influence cfPWV and thus obscure measurement of the true effect of acute sitting exposure on aortic arterial stiffness. Additional potential limitations include equipment expense, operator training requirements, operator bias, and the likelihood of poor signal quality in certain populations—such as those with obesity [12]. A measurement technique which overcomes these limitations would help to expand the field of SB research.

The goal of this research was to determine whether estimated pulse wave velocity (ePWV) is suitable for tracking the arterial stiffness response to acute prolonged sitting. ePWV, which is calculated using an equation incorporating age and mean arterial pressure (MAP) [13], moderately correlates with cfPWV [13,14]. In clinical settings, a single ePWV measurement can powerfully predict adverse health outcomes such as atrial fibrillation, CVD mortality, and all-cause mortality, both among healthy and clinical populations [15,16,17,18]. However, ePWV has not been used to estimate arterial stiffness responses to acute prolonged sitting exposures. Therefore, the aims of this secondary analysis [19] of overweight/obese adults were to: (1) determine the strength of the associations of baseline ePWV with cfPWV and carotid–radial PWV (crPWV); and (2) compare the ePWV, cfPWV, and crPWV responses to prolonged sitting during an 8 h simulated workday.

## 2. Materials and Methods

This study is reported in accordance with Consolidated Standards of Reporting Trials (CONSORT) guidelines [20]. All study procedures were approved by the human research protection office at the University of Pittsburgh (PRO16040501), and all participants submitted written informed consent.

### 2.1. Participants

We recruited middle-aged working adults who met the criteria: (1) aged 20 to 65 years; (2) systolic blood pressure (SBP) of 120 to 159 mmHg and/or diastolic blood pressure [DBP] of 80 to 99 mmHg); and (3) body mass index (BMI) of 25.0 to 40.0 kg/m^2^. Exclusion criteria included: (1) recent (<6 months) cardiovascular event; (2) use of cardiometabolic medications; (3) active treatment for a chronic condition; (4) current participation in a weight loss intervention; (5) pregnant in the past six months or breastfeeding in the past three months; (5) current smoker; and (7) engagement in ≥90 min/week moderate-to-vigorous-intensity physical activity over past three months.

### 2.2. Experimental Design and Overview

**Figure 1:**
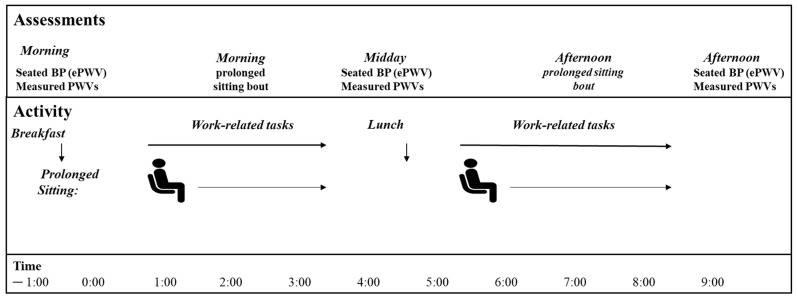
Experimental procedure. Participants arrived at our laboratory between 7:00 a.m. and 7:30 a.m. and performed a continuous uninterrupted prolonged sitting session. Blood pressure (BP) and measured pulse wave velocity (PWV) were completed in the morning, midday, and afternoon. Standardized meals (breakfast and lunch) were provided after morning and midday measurements.

This secondary analysis was conducted using data from a single condition in a randomized cross-over trial that examined the effects of two prolonged sitting sessions (i.e., with or without breaking up prolonged sitting with intermittent standing) on blood pressure and measured cfPWV and crPWV [19]. For the purpose of the current paper, we only used the data from the simulated workday session that involved prolonged sitting without breaking up with intermittent standing (see Figure 1). Participants arrived at the laboratory (University of Pittsburgh) between 7:00 and 7:30 a.m. having abstained from food, caffeine, and nicotine for 12 h and exercise and alcohol for 24 h. A standardized (10–15% protein, 25–30% fat, and 55–60% carbohydrate) breakfast and lunch were prepared and consumed to fulfill 30% of participants’ daily calorific need at each meal.

The experimental session included prolonged sitting, with blood pressure (BP) and PWV (cfPWV and crPWV) measurements collected in the morning (baseline), midday, and afternoon (post). The session began with participants resting in a seated posture for 10 min, followed by BP measurements. PWV measurements were then collected following 10 min of supine rest. Participants then returned to the seated posture to begin the simulated workday. Participants were required to complete personal, job-related, desk-based work while sitting at the desk during the sitting periods, which simulated an 8 h workday. Restroom breaks could be taken as needed, but other breaks to the prolonged sitting were not allowed.

### 2.3. Measurements

Age, sex, race, and education were self-reported using standard questionnaires. To calculate BMI [body weight (kg)/body height (m)^2^)], body weight was measured using a digital scale (WB-110A; Tanita, Japan) and height using a wall-mounted stadiometer (Perspective Enterprises, Portage, MI, USA).

#### 2.3.1. BP

Brachial BP was measured using a validated oscillometric device (HEM-705, Omron Healthcare, Inc., Lake Forest, IL, USA) [21]. A Gulick tape measure was utilized to measure the participant’s arm circumference to determine the proper BP cuff size. Before BP measurements were performed, participants rested in a chair for at least 10 min with arm supported at heart level, back supported, and feet resting flat on the floor [22]. Next, a BP measurement was taken from both arms. Following a 1 min interval, a second BP measurement was taken from the arm that had the higher initial BP. The two measurements on the higher arm were then averaged. This arm with the higher initial BP was used for subsequent measurements. MAP was calculated as DBP + 0.4 × (SBP−DBP) [13].

#### 2.3.2. Pulse Wave Velocity

Each PWV (cfPWV and crPWV) was calculated by dividing arterial path length (*D*) by the pulse transit time (PTT) between the proximal and distal arterial sites. For cfPWV, *D* was calculated by subtracting the linear distance between the sternal notch and carotid artery site from the linear distance from the sternal notch to the femoral artery site. For crPWV, *D* was measured as the linear distance between the carotid artery and radial artery sites. In accordance with guidelines [23], PTT was measured using tonometric sensors (Complior Analyse, ALAM Medical, Vincennes, France) to simultaneously record pulse pressure waveforms at the three arterial sites (carotid, radial, femoral). An inherent proprietary algorithm calculated PTT as the time from the foot of the proximal pressure waveform to the foot of the distal pressure waveform. Measurements were made in triplicate, with each scan encompassing 10 pressure waveforms. Each PWV measurement was checked for quality, and high-quality measurements (coefficient of variation within scan <10% across waveforms) were averaged for a single value per participant. Using this protocol, our laboratory has excellent inter-operator (0.91) and intra-operator (0.94–0.98) intra-class correlation coefficients for cfPWV.

#### 2.3.3. Estimated Pulse Wave Velocity

ePWV was calculated using the published formula [13] as follows: ePWV = 9.587 − 0.402 × age + 4.560 × 10^−3^ × age^2^ − 2.621 × 10^−5^ × age^2^ × MAP + 3.176 × 10^−3^ × age × MAP − 1.832 × 10^−2^ × MAP.

### 2.4. Statistical Approach

Stata version 17 (StataCorp, LLC, College Station, TX, USA) was utilized for all data analyses. Statistical significance was set at *p* < 0.05. Participant characteristics and baseline (morning) BP and PWV were summarized as means and standard deviations or frequencies and percentages. To test aim 1, Pearson correlation coefficients (*r*) were used to determine the strength of association of baseline ePWV with cfPWV and crPWV. The strength was rated as weak (*r ≤* 0.3), moderate (*r ≤* 0.6), or strong (*r ≥* 0.7) using conventional thresholds [24]. For addressing our second aim, separate generalized linear models estimated the ePWV, cfPWV, and crPWV response to the simulated workday. These models estimated responses first without adjustment, with adjustment for MAP or BMI, and with adjustment for both MAP and BMI [25,26,27]. Using Cohen’s *d*, effect sizes of the responses to prolonged sitting were interpreted with respect to meaningfulness as small (*d* = 0.2), medium (*d* = 0.5), or large (*d* = 0.8) [28].

## 3. Results

### 3.1. Participants

Table 1 displays the general characteristics of our participants (*n* = 25). On average, the participants were highly educated and middle-aged, had elevated BMI and SBP per the inclusion criteria, and the majority were male (64%) and identified as non-Hispanic white (80%).

### 3.2. Aim 1: Baseline Associations

As reported in Table 2, baseline (morning) ePWV was strongly correlated with cfPWV and moderately correlated with crPWV.

### 3.3. Aim 2: Response to Prolonged Sitting

As reported in Table 3, cfPWV significantly increased over time without and with adjustment for MAP and/or BMI (*d* ranged from 0.40 to 0.40). In contrast, ePWV minimally and non-significantly increased over time either without or with adjustment for MAP and/or BMI (*d* ranged from 0.01 to 0.09). Similarly, crPWV did not significantly increase over time either without or with adjustment for MAP and/or BMI (*d* ranged from 0.11 to 0.16, respectively).

## 4. Discussion

At baseline, ePWV was strongly associated with cfPWV and moderately associated with crPWV. However, while cfPWV moderately increased in response to the simulated workday of mostly prolonged sitting, ePWV along with crPWV did not significantly change.

### 4.1. Limitations and Strengths

A few potential limitations should also be considered when interpreting our results. Our sample was selected for certain eligibility characteristics based on the aims of the original randomized crossover trial, and results may not be generalizable to a broader population. Similarly, most of the sample (80%) was non-Hispanic white adults, which could further limit generalizability. In addition, previous studies found that individuals with obesity are at higher risk for developing metabolic syndrome [29], which can influence vascular structure [26] and functions, including PWV [27]. However, data on metabolic syndrome was not obtained for this secondary analysis due to the nature of the parent study [19], further limiting the generalizability of our findings. Still, several strengths of this study should also be considered. Namely, gold standard assessments of PWV and BP were used. Additionally, our simulated workday had high ecological validity, as it was designed to simulate a typical workday. Our protocol was of longer duration compared to previous prolonged sitting studies (>7 h vs. ≤3 h) [9,30,31] and may better represent responses across a typical workday.

### 4.2. Comparison to the Literature: Aim 1

We observed a strong correlation between baseline ePWV and cfPWV (*r* = 0.70) and a moderate correlation between ePWV and crPWV (*r* = 0.55) among American adults with mild-to-moderately elevated BP who were overweight or obese. Our findings are largely in agreement with studies reporting a weak-to-moderate correlation (*r* ranged from 0.35 to 0.66) between ePWV and cfPWV in Danish and French [13], Australian [14], and American [32] adults. A moderate correlation (*r* = 0.67) has also been reported between ePWV and invasive aortic PWV in Australian adults [14]. Similarly, a moderate correlation (*r* = 0.66) between ePWV and brachial–ankle PWV (baPWV) was reported in Chinese adults [33]. Of note, some of the observed correlations were lower than one would expect from highly related measures (*r* < 7). This lower correlation may be explained by different vascular segments for each measure (specific details are in the following section) as well as differences in the population that was used to develop the ePWV measure vs. the populations in the subsequent studies, including our own. Taken together, these findings suggest that ePWV, at least in stable (i.e., non-acute) conditions, acceptably correlates with PWV measured from different vascular segments and in diverse ethnic/racial groups. These findings are promising, as ePWV powerfully predicted future CVD and mortality in large cohorts [13,15,16,17].

### 4.3. Comparison to the Literature: Aim 2

In response to the simulated workday, while cfPWV moderately increased (*d* = 0.40), neither ePWV (*d* = 0.01) nor crPWV (*d* = 0.11) significantly changed. As such, while ePWV was found to be a useful and powerful tool for predicting future CVD and mortality in clinical settings [13,15,16,17] under stable conditions, ePWV may not be suited to tracking acute changes in arterial stiffness in experimental settings. The distinct origins of ePWV and cfPWV may help to explain the divergent observations. While cfPWV is measured across the central vasculature (carotid–femoral segment), ePWV is calculated using a measure taken from peripheral vasculature (i.e., brachial MAP). We have reported that prolonged sitting has more pronounced influences on the central (i.e., cfPWV) compared to upper peripheral vasculature (i.e., crPWV) [10]. Consequently, it might be hypothesized that the direction and magnitude of ePWV and crPWV responses are more likely to agree than ePWV and cfPWV responses. We cannot confirm this from the current findings because, as expected, crPWV did not significantly increase in response to the simulated workday, and we cannot mathematically compare two non-responses. This finding underscores that, in contrast to cfPWV, crPWV does not appear to capture the vascular effects of prolonged sitting.

## 5. Conclusions

The goal of this research was to determine whether ePWV is suitable for tracking the arterial stiffness response to acute prolonged sitting. At baseline, ePWV was moderately associated with cfPWV and crPWV. However, while cfPWV moderately increased in response to the simulated workday, ePWV and crPWV did not change significantly. These preliminary data indicate that ePWV does not capture acute vascular responses that are evident when cfPWV is measured by tonometry. Therefore, ePWV does not appear to be suitable as a surrogate measure, and cfPWV should be utilized to capture acute changes in vascular stiffness in experimental sitting studies. We cannot rule out the potential development of an ePWV equation that can track acute arterial stiffness response. However, the measurement of MAP from the periphery, at least without additional information, may be insufficient for such an equation.

## Figures and Tables

**Table 1:** Participant characteristics (*n* = 25).

Characteristic	Mean (SD) or *n* (%)
Age (years)	42.2 (11.7)
BMI (kg/m^2^)	31.9 (5.0)
Sex	
Male	16 (64%)
Female	9 (36%)
Race	
Non-Hispanic White	20 (80%)
Non-Hispanic Black	2 (8%)
Hispanic White	1 (4%)
Asian	2 (8%)
Education	
Post Graduate Degree	15 (60%)
College Graduate	5 (20%)
High School Graduate	5 (20%)
Baseline BP	
SBP (mmHg)	124.0 (12.9)
DBP (mmHg)	76.6 (10.9)
MAP (mmHg)	95.5 (11.3)
Baseline PWV	
Epwv	7.6 (1.5)
cfPWV	7.6 (1.2)
crPWV	9.3 (1.6)

BMI: body mass index; cf: carotid–femoral; cr: carotid–radial; DBP: diastolic blood pressure; e: estimated; MAP: mean arterial pressure; *n*: number; PWV: pulse wave velocity; SBP: systolic blood pressure; SD: standard deviation.

**Table 2:** Correlations between baseline estimated (ePWV) with carotid–femoral (cfPWV) and carotid–radial (crPWV) pulse wave velocities (PWV).

Outcome	*r*	LCI	UCI	*p*
cfPWV	0.70	0.54	0.80	0.0001
crPWV	0.55	0.36	0.70	0.0043

LCI, lower confidence interval; UCI, upper confidence interval.

**Table 3:** Estimated (ePWV) carotid–femoral (cfPWV) and carotid–radial (crPWV) pulse wave velocity (PWV) responses to a simulated workday with prolonged sitting (*n* = 25).

Outcome	Model	*β* (SE)	*p*	*d*
ePWV (m/s)	Unadjusted	0.14 (0.09)	0.135	0.09
	Adjusted for MAP	−0.02 (0.01)	0.259	0.01
	Adjusted for BMI	0.14 (0.09)	0.135	0.09
	Adjusted for MAP and BMI	−0.02 (0.01)	0.257	0.01
cfPWV (m/s)	Unadjusted	0.52 (0.20)	0.010	0.43
	Adjusted for MAP	0.48 (0.21)	0.021	0.40
	Adjusted for BMI	0.52 (0.20)	0.010	0.43
	Adjusted for MAP and BMI	0.48 (0.21)	0.021	0.40
crPWV (m/s)	Unadjusted	0.25 (0.23)	0.273	0.16
	Adjusted for MAP	0.18 (0.24)	0.437	0.11
	Adjusted for BMI	0.25 (0.23)	0.273	0.16
	Adjusted for MAP and BMI	0.18 (0.24)	0.434	0.11

*β*: beta coefficient; *d*: Cohen’s *d* (*β*/standard deviations of baseline values); m/s: meter per second; MAP: mean arterial pressure; SE: standard error.

## Data Availability

The data presented in this study are available in this article.
